# Implementation of the Video Assistant Referee (VAR) as a Career Change-Event: The Israeli Premier League Case Study

**DOI:** 10.3389/fpsyg.2020.564855

**Published:** 2020-10-30

**Authors:** Roy David Samuel, Yair Galily, Edson Filho, Gershon Tenenbaum

**Affiliations:** ^1^Interdisciplinary Center Herzliya, Herzliya, Israel; ^2^Wheelock College of Education and Human Development Boston University, Boston, MA, United States

**Keywords:** football, officials, decision-making, video replay, transition, technology

## Abstract

The inclusion of the video assistant referee (VAR) in the Laws of the Game [International Football Association Board (IFAB)] reflects a historic action in the world of soccer. The VAR was designed to reduce critical errors in soccer referees’ decision-making (DM), thereby increasing the social perceptions of justice. From the referees’ perspective, the implementation of the VAR represents a technical–technological career change-event. This case study adopted an intrinsic mixed-methods methodology to investigate the implementation of the VAR system within the Israeli Premier League context. The results indicated that the initial VAR performance rates of the Israeli referees were not high compared with data from other countries (e.g., Italy). The Israeli referees perceived the VAR implementation as a moderate change-event in their careers. The largest effects were in pre-match preparation, players’ management, public perception, and DM. The referees felt that their perceived pressure during the matches decreased. This change-event produced off-field demands mostly related to the educational process and on-field demands mainly related to developing VAR proficiency. The referees also experienced off-field (e.g., lack of clear goals and rewards system) and on-field barriers (e.g., errors of the VARs). To effectively cope with these new demands and barriers, most of them initially consulted with others and then made a decision to apply all necessary adjustments in response to the new situation. They also received considerable support yet perceived their cooperation with the professional committee as moderate. The referees showed some professional improvements; still they experienced scrutiny from the clubs, the media, and the Referee Union. Thus, three of them perceived the outcome of this change process favorably, four naturally, and four negatively. The discussion presents reflections of these findings in light of the recently emerging literature on technological officiating aids in sport. Recommendations are provided for referee unions who aspire to integrate the VAR system into their operation.

## Introduction

I was left with the decision I had taken with no independent evidence that I’d got it wrong other than a gut feeling, and I was just hoping that Ronaldo would miss the penalty. But he didn’t (Bradbury, 2020).

This quote of former English Premier League referee, Howard Webb, reflects one of the key aspects of soccer (association football) refereeing, namely, decision-making (DM). In this context, the inclusion of the video assistant referee (VAR) in the Laws of the Game (International Football Association Board [IFAB], 2018b) and its introduction in the 2018 World Cup represent a historic action in the world of soccer (Simón, 2020). This system is aimed to reduce critical DM errors in soccer refereeing, focusing on four areas: approval/disapproval of goals, penalty decisions, direct red card decisions, and mistaken identity in awarding a red or yellow card (International Football Association Board [IFAB], 2017). Within these four areas, the VAR system reviews the various match events, conducting a silent check. In case that the video referee identifies a potential clear error, s/he can then communicate with the on-field head referee, who in turn can (a) change the call on the advice of the VAR, or (b) conduct an *on-field review* (OFR), using a designated monitor station. There are specific protocols for the usage of the system, including when intervention is allowed, how to signal that an intervention is in order, and various player management rules (International Football Association Board [IFAB], 2017). In terms of the refereeing DM sequence, VAR intervention appears at the end of the process, when the on-field referee has already made a decision and receives external feedback from the refereeing team. S/he is then faced by a subsequent decision, namely, to keep or change the original decision (Samuel et al., 2020b).

Arias et al. (2011) reviewed the literature on rule modification in sport and identified that most rule modifications were intended to improve sports performance, attract spectators, and attend to commercial pressures and interests. The VAR system rule modification was mainly due to pressure from club owners and the media to improve refereeing performance and, consequently, justice in the game (Simón, 2020).

The VAR is a technological system that includes a human factor (i.e., the video referee and assistant referee, the operator), a technological factor (the video system, the audio system), a human–technological interface (Sánchez Cid and García García, 2020), and a social–ethical factor (e.g., referee team dynamics, perceptions of fairness and justice; de Dios Crespo, 2020). As such, its introduction presented major modifications in the way referees train, prepare for, and officiate matches (Armenteros and Webb, 2020; Fernández Ruiz et al., 2020) as well as in refereeing psychology (de la Vega and Fuentes, 2020). Furthermore, Kolbinger and Lames (2017) reviewed the literature regarding the use of technological officiating aids in various game sports. They identified seven major issues: the underlying phenomena, usage patterns, accuracy, standard of review, influence on the nature of the game, material as well as immaterial costs, and the amount of authority that is granted to the officiating aid. It could be argued that VAR is a technological aid with important ramifications for all these dimensions.

Among semi-elite Australian rugby league referees, for example, the inclusion of the video referee technology had led to various stressors pertaining to training, DM, media, coaches and players, and communication issues (Baldwin, 2014). These changes resulted in significant challenges in terms of referees’ coping and motivation. Yet, currently, there is a scarcity of scientific publications concerning the usefulness of the VAR system, as well as its potential influence on referees’ motivation, performance, and development (Samuel et al., 2020b). The present case study, therefore, examined the implementation of the VAR system as a technical–technological change-event within Israeli referees’ careers (Samuel et al., 2017).

### The Emergence of the Video Assistant Referee System

The VAR system was initially trialed by various national associations, like the Italian Serie A (Simón, 2020) and the German Bundesliga (Kolbinger, 2020). An analysis that was based on these initial trials (i.e., 804 matches) concluded that, in most of the matches (68.8%), there was no review and, on average, in every three matches, there was one clear and obvious error. The system improved the total accuracy in key match decisions (i.e., in the four categories that VAR intervenes in) from 93 to 98.9%. Moreover, in 8% of all matches, the VAR had a decisive impact on the outcome of the match (International Football Association Board [IFAB], 2018b). As a result of these positive analysis outcomes, on March 3, 2018, the IFAB wrote the VARs into the Laws of the Game on a permanent basis, presenting its decision as a “historic step for greater fairness in football” (International Football Association Board [IFAB], 2018a).

An analysis of 1,024 matches played in the Italian Serie A and the German Bundesliga league during the seasons before and after the implementation of the VAR system revealed a decrease in the number of offsides, fouls, and yellow cards after the implementation of the VAR (Lago-Peñas et al., 2019). There was an increase in the number of minutes added to the playing time in the first half and the full game, but not in the second half. Finally, there were no significant changes in the number of penalties or red cards per match (Lago-Peñas et al., 2019). It was further proposed that using technological officiating aids in sport might undermine the authority of the referee, interfere with the flow of the match, and even create injustice when on-field decisions are overruled by the video review (Kolbinger and Lames, 2017). Moreover, better visual coverage of the field and match events can result in detecting more minor incidents, previously overlooked by referees, players, and fans. This can be considered as one of the potential threats of including such a technology aid (Kolbinger and Lames, 2017; de Dios Crespo, 2020).

Another issue that might raise concern is the effectiveness of communication between the on-field referee and the VAR through the headset (Baldwin, 2014; Sánchez Cid and García García, 2020). The referee is expected to trust the judgment of the VAR in critical match incidents and his/her professional skills in deciding on the correct decision. This might entail a significant change in the authority of the head referee to officiate the match autonomously. Considering the role of the VAR, this task is new, requiring the referees to engage in video DM, in most cases out of context and under much pressure and public scrutiny.

### Video Assistant Referee as a Career Change-Event

The scheme of change for sport psychology practice (SCSPP; Samuel and Tenenbaum, 2011a) is a conceptual framework describing typical characteristics of change processes in sport performers’ careers. The change process begins with the emergence of a change-event that disrupts the career *status quo* and creates emotional and cognitive imbalance. Each change-event is characterized by unique demands (i.e., perceived challenges associated with the various emerged changes). Performers then evaluate the meaning and significance of the event in the context of their careers, considering existing resources of coping and potential solutions. Change-events are characterized by unique *emotional profiles* pertaining to perceived significance, perceived severity, perception of others, emotional and cognitive reactions, and perceived control over the situation. This appraisal process leads to active decisions; initially, *a strategic decision* concerning preliminary response to the new situation (i.e., deny/ignore it, cope independently, consult with others, consult with a sport psychologist) and a subsequent decision to avoid change or to *a decision to change* (i.e., apply all required modifications to effectively cope with the new situation). It is assumed that if referees decide to change, and also have a possibility to implement the change, they will feel efficient in their coping efforts, in control over the situation, and accept responsibility for initiating the change. They would then better cope with the change process barriers (i.e., factors that debilitate effective adaptation). As a result, the change process outcome will be perceived more favorably (Samuel and Tenenbaum, 2011a; Samuel et al., 2017; Samuel, 2019).

Previous research indicated a highly dynamic soccer refereeing career, comprising of various transitions and change-events (Slack et al., 2013; Samuel et al., 2017; Samuel, 2019). Using the SCSPP as a conceptual framework, Samuel et al. (2017) examined the career experiences of 154 Israeli referees from several professional levels. The referees experienced over 10 types of change-events in their careers, including *a transition to a higher league*, *excelling in a big match*, and *a very poor performance or a decision error in a big match*. Half of the sample reported experiencing *the initiation of the communication system*, which could be classified as a technical–technological modification. This study further indicated that most referees made an initial strategic decision to consult with others in response to the initiation of a change-event and a subsequent decision to change.

The emergence of the VAR system in soccer refereeing can be conceptualized as a quasi-normative transition (i.e., applies to a selected group of elite referees; Stambulova and Samuel, 2020). The VAR system challenges referees to modify their training and performance. This might influence their motivation for refereeing directly and indirectly through effects on performance and professional and public credibility and reputation. Anecdotal evidence suggests a mixture of opinions regarding the usefulness and necessity of the VAR system among referee union leaders (Simón, 2020). In this context, VAR implementation was challenging in most relevant countries, emphasizing the difficulty in integrating such a major change.

### Study Purpose and Objectives

How do soccer referees perceive and respond to the implementation of the VAR system? How does this system influence their training, performance, and career status? How do they cope with these changes? To date, no empirical study examined how referees perceive the integration of the VAR system as a change-event in the context of their careers. As this system might induce various types of changes in referees’ training, performance, and career status, it is important to study its potential effects on referees’ careers. Therefore, the present study used the SCSPP (Samuel and Tenenbaum, 2011a) framework to examine elite referees’ perceptions and responses to this change-event, as well as their coping efforts (including conscious coping decisions) and support resources. As each country has its own unique referee union structure and cultural environment and a specific process in which VAR was initiated, it is important to carefully consider context. Thus, we applied a case study methodology to examine the implementation of the VAR system within the context of the Israeli Referee Union and the Israeli Premier League—“Ligat Ha’Al.” We begin by presenting the official data concerning the general VAR-related performance of the Israeli referees. Subsequently, our focus is on understanding the referees’: (a) perceptions of this change-event, (b) evaluations of the major areas of change (i.e., demands and barriers), (c) conscious decisions and coping, (d) support resources, and (d) the change process outcomes (including effects on motivation).

## Materials and Methods

### Design

An intrinsic mixed-methods case study methodology was applied (Yin, 2014; Hodge and Sharp, 2016). Specifically, a pragmatic approach was adopted with hopes to holistically describe the referees’ change process in their unique context (Patton, 2002; Sparkes, 2015). Qualitative data (i.e., interview notes, referees’ feedback) were collected over a 19-month period, lasting from the beginning of the VAR implementation process (September 2018) to the end of the regular season (March 2020). In addition, quantitative data [i.e., the Change-Event Inventory (CEI); Samuel and Tenenbaum, 2011b] were collected in a single administration point, during January–February 2020 (i.e., middle of the 2019–2020 season). This time point was selected to ensure the referees already had sufficient experience with the VAR system but were still experiencing the change process (i.e., they were still attempting to adjust to the VAR). A similar approach was previously used to examine referees’ change process to the Premier League (Samuel, 2019). The referees’ experiences regarding the implementation of the VAR system were analyzed within the Israeli Premier League context as they were advancing throughout their change processes. Data sources complemented each other.

### Case Study Context—Video Assistant Referee in the Israeli Premier League

Following the IFAB’s decision to implement the VAR in various federations beginning of March 2018 and the successful 2018 World Cup, the major European countries integrated VAR in their regular leagues. Israel is affiliated to the Union of European Football Associations (UEFA). The Israel Football Association, together with the Israeli Professional Football Leagues Directory, has therefore, began considering implementing a VAR project in Israel. The Directory received a green light from the teams and a financial assurance for 5 years’ term. In fact, Israel was somewhat a pioneer in this context, as one of the small–medium size countries to implement such a complex project. In September 2018, a public announcement was made that the VAR would already be integrated in the same season’s playoff matches beginning mid-March 2019. This created a very tight schedule for such a complex implementation project—6 months compared with the IFAB’s recommended 12-month period—resulting in high pressure and a very focused process. Two project managers were appointed in charge of setting the technological infrastructure as well as the professional instruction and certification. As the VAR project was novel, the professional manager had to individually strategize the implementation process, including the comprising of an instruction program to align with the IFAB’s strict certification demands. On top of certifying the referees, VARs, assistant VARs, and operators, there was a need to technologically certify all hosting stadiums to receive official IFAB certifications. Furthermore, a training area was required to be modified to include two simulator rooms and an instruction classroom.

The theoretical and practical instruction of the referees during the active league season was challenging, as they were preoccupied with training and match officiating. There was a strict learning protocol that each referee went through, including (a) theoretical knowledge of the VAR system and protocol, (b) an offline simulator of clips and then full matches, and (c) live simulations. As a final step, the referees experienced offline performance in real Premier League matches. In total, the referees engaged in 45 h of instruction. Then, they initiated a pilot phase in the Premier League upper playoff of the 2018–2019 season (i.e., 30 matches in total). As part of the continued instruction, the project manager provided feedback to the VARs following each performance, including analysis of technical performance and teamwork between the VAR and the operator.

### Participants

The participants were 11 male elite referees from the Israeli Premier League (*M*_age_ = 34.54 years, *SD* = 6.23), representing 65% of the designated population. Their refereeing experience ranged from 10 to 25 years (*M* = 18.14 years, *SD* = 5.41). Three of the referees were relatively new to the Premier League (i.e., 1–3 seasons), two had moderate experience (i.e., four seasons), and six were seasoned referees.

### Procedure

We followed the American Psychological Association’s ethical guidelines concerning the formulation of a case study with existing clients (APA, 2017). Specifically, genuine voluntariness was maintained as the participants could choose to not participate in this case study or withdraw from it at any point. Also, they could decide whether to exclude any of their related information. In addition, the participants provided their written informed consent. Finally, the first author engaged in an open dialogue with the participants concerning the formulation and presentation of the case study data and debriefed them concerning the results and conclusions (APA, 2017). As the data were obtained as part of an ongoing consultation process, no institutional ethical approval was required.

Initially, the participants were informed of the study’s overarching purpose, i.e., a case study concerning professional referees’ experiences with the newly introduced VAR system. They provided their preliminary verbal consent on a voluntary basis. Qualitative data sources (e.g., interview notes, open-ended inventory data) were collected as part of an ongoing consultation process involving the first author and the referees. Additionally, the VAR project manager was contacted and asked to provide his account of the VAR implementation process and the official data on VAR-related performance. Also, the first author visited the VAR instruction center and the Video Operations Room (VOR) during a live match. Moreover, the referees were provided an explanation as to how to complete the CEI; they were asked to reflect on their individual VAR implementation experience and to complete the CEI at home. Upon manuscript completion, all participants received a copy of it and provided their informed consent for their anonymized data to be presented. They were asked to read the manuscript, providing their insights and suggestions for modifications. Data collection and analysis ensured, to the possible degree, the anonymity and confidentiality of the participants’ information.

### Data Collection and Treatment

#### Qualitative Data Collection

There were three main qualitative data sources. First, the participants received sport psychology support as an ongoing service provided by the Israel Referee Union. In these semimonthly 1-h sessions, the referees consulted with the first author on an array of issues pertaining to their refereeing engagement, including psychological preparation for matches and match analysis (see Samuel, 2015), organizational stress, and career management (see Samuel et al., 2017; Samuel, 2019). Since the introduction of the VAR system, much attention was dedicated to assessing the referees’ perceptions of the system, how it impacted them in terms of performance and organizational demands, and how to effectively cope with this change-event. During these sessions, interview notes were taken pertaining to main ideas and thoughts, direct quotes of the referees’ words, as well as the consultant’s own interpretations. The first author then read and reread these notes (i.e., familiarization), identifying all references made to the VAR system and collecting them into a single file (see Patton, 2002). This practice was used in previous change-event studies with mixed-methods designs (e.g., Samuel, 2019).

Second, as part of completing a measurement related to their transition experiences, the referees were asked to freely elaborate in writing on (1) the new demands (i.e., what an athlete wants/ought to achieve in going through the transition, Stambulova and Samuel, 2020) associated with officiating with the VAR system in professional, psychological, occupational, time, and training aspects; (2) the barriers and difficulties experienced throughout the VAR implementation process; (3) their coping efforts when addressing this change-event; and (4) any additional comments and recommendations they have. Finally, for the purpose of this study only, the VAR project manager of the Israel Referee Union was asked to provide a detailed internal report on the implementation process, including the decision to implement, the professional demands, the instructional process, main barriers, and the referees’ performances. In addition, the VAR project manager provided the official data concerning all matches officiated with the VAR system in Israel (i.e., for all Premier League referees) throughout the 2018–2019 Pilot Phase and two thirds of the 2019–2020 Season.

#### Quantitative Data Measurement

To assess the referees’ change process, they completed a *modified Change-Event Inventory* (Samuel and Tenenbaum, 2011b). The original CEI measures change-event experiences in a retrospective manner using a three-section format: (a) demographic information; (b) perception of and reaction to a change-event (i.e., the implementation of the VAR system), measured by 13 two-item Likert-type subscales (e.g., perceived significance of the event, perceived emotional severity of the event, perceived control over the event), ranging from 1 to 5 (1, *not at all/very negative*; *3*, *moderate/neutral*; *5*, *very much/very positive*); and (c) coping-related DM and availability of support resources. Previous research on a heterogeneous sample of competitive athletes indicated adequate psychometric properties of the CEI, including temporal stability, internal consistency (i.e., all Cronbach’s α ranged between 0.68 and 0.89), and internal factorial structure (Samuel and Tenenbaum, 2011b).

For the purpose of this study, as there is no existing VAR-related measure, the CEI was modified to capture the referees’ VAR implementation experience, and additional items were added to the inventory. First, a VAR-specific section was added, which included items assessing the participants’ enjoyment of refereeing with the VAR system and how they evaluate their performance since it was introduced. Also, the degree in which they evaluate the VAR’s impact in 15 areas related to performance (e.g., running patterns, DM, and game management), referees’ emotional response (e.g., changes in self-confidence and in stress levels), and in organizational and public perceptions. Second, two items pertaining to satisfaction from the cooperation with the professional committee concerning the VAR were included in the second section of the inventory. Most two-item subscales indicated adequate internal consistency indices (i.e., α = 0.67–0.91), despite the small sample size. Low reliability indices (i.e., α ≥ 0.57) were found, however, in *past experience in similar events*, *satisfaction of coping*, *capacity for change*, *cognitive response*, and *the perception of others*.

### Data Analysis

In line with Sparkes (2015) recommendations concerning quantitative and qualitative data sources in mixed-methods case studies, all data sources were integrated into a coherent account of the soccer referees’ change process. Data were collected as part of psychological support services, so confidentiality considerations were pertinent (APA, 2017). Data are, therefore, presented with no identifying cues. The qualitative data were read several times. An indwelling posture was adopted by going through the data, immersing in it, and understanding the referees’ point of view from an empathetic position (Holt and Sparkes, 2001). The lengthy consultation notes as well as the referees’ open-ended inventory responses were summarized by identifying key events, thoughts, and issues pertaining to each referee’s experiences within his change process. The analysis then focused on the condensed summary report created for each referee, as well as the referee’s quantitative data. Yin (2014) suggested that the preferred strategy in a case study analysis is to follow a theoretical framework that accounts for “how” and “why” questions. Therefore, the SCSPP framework (Samuel and Tenenbaum, 2011a) was used to understand the referees’ change process. Each of the 11 referees was considered a *unit of analysis*, and attention was given to the individual unique experience as well as to the shared experiences. Specifically, the analysis focused on realizing the on- and off-field demands and barriers associated with the VAR implementation, as well as the referees’ perceptions of the change process in the context of their careers, their emotional and cognitive reactions, how they had coped with it (i.e., their DM, their coping efforts, and the existing available support), and the outcome of the change process in the context of their careers.

In addition, the quantitative data were analyzed using descriptive and inferential statistical procedures, including reliability indices, central tendency metrics, and correlations among variables. With a relatively small sample of 11 participants, we were limited in statistical power (Schweizer and Furley, 2016), and any generalization of the quantitative data should be considered with a degree of caution.

### Rigor and Quality of the Case Study

Evaluating the rigor of this study, we have adopted a relativist stance and created our list of criteria (Sparkes, 2015; Smith and McGannon, 2018). First, we believe this is *a worthy topic* (Tracy, 2014), as not much research effort was devoted to examining referees’ experiences with the VAR system. Moreover, the first author was well-positioned within the case study context, serving for many years as a sport psychologist of the Israel Referee Union. This experience facilitated the formation of clear research questions as well as the *credibility* of data collection and analysis (Patton, 1999; Tracy, 2014). The lead researcher also engaged in self-reflection, maintaining a critical mindset throughout the research process, including data collection and analysis. Still, we were aware of potential bias, and for this reason, the other team members were involved in the data analysis as “critical friends.” Specifically, the research team engaged in a process of reflexive dialogue concerning the interpretations of the data (Sparkes and Smith, 2014). *Triangulation* was conducted by applying various types of data sources (i.e., qualitative and quantitative; Patton, 1999). Using the SCSPP (Samuel and Tenenbaum, 2011a) as a conceptual framework to guide the data analysis further increased credibility. In the “Results” section, we present a *coherent* and *comprehensive* narrative of the referees’ experiences, in a unique context, by providing themes and associated direct quotes (i.e., a thick description; Patton, 2002). Finally, concerning the qualitative data, representational generalizability (Smith, 2018) was reached by recognizing similarities and differences to the results of previous athletic transition research. Using the SCSPP framework to analyze the referees’ experiences, analytical generalizability was produced (Smith, 2018). In line with Smith and McGannon’s (2018) recommendations, upon completion of the data analysis process, the manuscript was sent to the participants for *member reflections*. We asked them for their feelings and perspectives regarding the presented data and interpretation. The referees and the VAR project manager conveyed that the presented data were rich and accounted for their transitional experience. Noteworthy, this process of member reflections is also congruent with ethically sound research, as it allows participants to protect their well-being by identifying any misrepresentations of their data.

## Results

In this section, we initially provide data concerning the performance of the Israeli Premier League referee squad with the VAR system, as this information is important to contextualize this case study. Subsequently, we present the experiences (change process) of the study participants in detail, including perceptions, demands and barriers, decisions and coping, support resources, and change process outcomes.

### The Israeli Video Assistant Referee Experience

As part of the data collection, the Israeli VAR project manager provided the official refereeing performance data of all 212 matches officiated with the VAR system in Israel until March 1, 2020 (Table 1). This period included the VAR system Pilot Phase in the 2018–2019 season playoff and two thirds of the 2019–2020 season (i.e., which was abrupted due to the Coronavirus pandemic). During this period, all 17 Premier League referees had officiated with the VAR system 6–17 matches each (*M* = 12.40, *SD* = 3.92). As shown in Table 1, across all 212 matches, there were 89 critical match errors made by the on-field referees. More specifically, during only two thirds of the 2019–2020 season (i.e., 182 matches in total), there were 81 critical errors. This relatively high error rate reflected a significant (*p* < 0.05) increase in the number of critical errors made by the on-field referees compared with the 2018–2019 season (69 critical errors), the 2017–2018 season (49 critical errors), and the 2016–2017 season (52 errors).

**TABLE 1:** The Israeli referees’ performance with the VAR system throughout the 2018–2019 pilot phase and two thirds of the 2019–2020 season.

**Factor**	**30 pilot matches**	**Matches 1–45**	**Matches 46–90**	**Matches 91–135**	**Matches 136–182**
Overall critical errors	8	21	22	19	19
Rectified on-field critical errors	7	13	15	16	18
Correct VAR interventions	7	17	19	17	18
Incorrect VAR interventions	4	6	5	3	2
Missing VAR interventions	1	4	3	2	1
Total OFRs	10	21	20	14	15
Correct OFRs	6	15	15	11	13
Incorrect OFRs	4	6	5	3	2
Match time suspension during VAR check	199 s	198 s	150 s	164 s	155 s
Total match time suspension	116 s	108 s	92 s	82 s	79 s

*OFR, on-field review; VAR, video assistant referee.*

Sixty-nine of the 89 critical errors performed by the on-field referees (77.5%) were correctly rectified due to VAR intervention. In total, the referees conducted 80 on-field reviews (OFRs), which translate into one OFR every 2.65 matches on average. They modified their original decisions in 55 of the cases (68.75%). In nine of the cases, the on-field referee did not modify his original decision and was incorrect, whereas in three of the cases, the referee wrongly modified his original decision due to a VAR error. Furthermore, across all matches, the VARs intervened 98 times. As can be seen in Table 1, in 78 cases (79.60%), this intervention was accurate, whereas in 20 cases (20.40%), the intervention was incorrect. Also, in 11 cases, the VAR did not intervene when it should have.

### Referees’ Perceptions of the Video Assistant Referee

**FIGURE 1:**
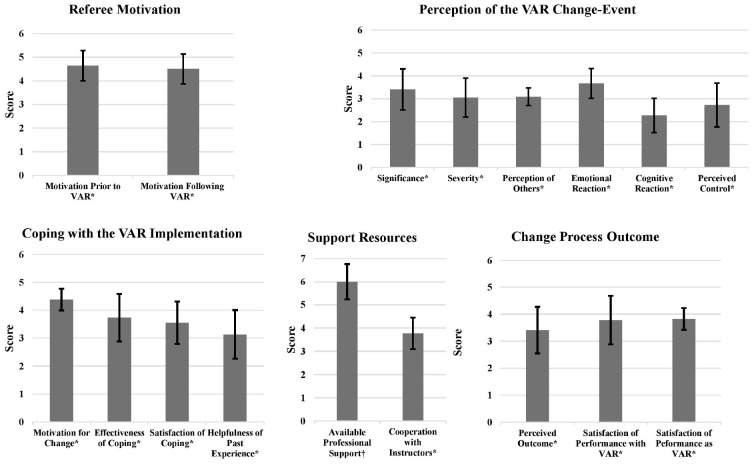
Means and SDs for the referees’ motivation **(upper left panel)**, perception of the video assistant referee (VAR) change-event **(upper right panel)**, coping with the VAR change-event **(lower left panel)**, support resources **(lower middle panel)**, and change process outcome **(lower right panel)**. *Scale is 1–5, ^†^Scale is 1–7.

The referees’ perceptions of the VAR system are presented in Table 2 and in Figure 1. As shown in Table 2, the referees had initially wished the system to be implemented in the Israeli league. Yet, their current evaluation of enjoyment using the system and their appreciation of the system was somewhat moderate. These quantitative data correspond to the qualitative data concerning the progress of the VAR implementation in the Israeli context. In the beginning of the process, the system was perceived as new and innovative, and most attention was given to its initial learning and implementation through the IFAB instruction protocol. Thus, most of the referees were appreciative about implementing the system. There was also strong pressure from the clubs and fans to implement it, as they perceived the system as a professional tool to facilitate accuracy and, thereby, justice in refereeing. However, as the system got integrated in the 2018–2019 season playoff matches, the referees were still inexperienced, and therefore, criticism of the referees’ performance had begun to emerge. This had significantly amplified in the 2019–2020 season. The referees felt that they had invested much effort in learning to officiate with VAR, yet still their performance was scrutinized. This situation had led to some negative change in the way the referees perceived the system, as one of them explained:

**TABLE 2:** Descriptive statistics of VAR perceptions and associated refereeing modifications.

**Perceptions**	***N***	**Min**	**Max**	***M* (*SD*)**
Wishing the VAR system to be implemented in Israel	11	3.00	5.00	4.37 (0.81)
Enjoying officiating with VAR as on-field referees	11	3.00	5.00	3.91 (0.83)
Enjoying officiating as VARs	11	2.00	5.00	3.78 (1.10)
Appreciating the VAR system as facilitating officiating	11	2.00	5.00	3.82 (0.87)
**The influence of VAR on:**				
Running pattern and locations	11	1.00	2.00	1.18 (0.40)
Foul evaluation	11	1.00	4.00	2.18 (0.87)
Decision making	11	1.00	4.00	2.54 (0.82)
Referee team communication	11	1.00	4.00	2.45 (1.21)
Players management and communication	11	1.00	5.00	2.64 (1.29)
Focus throughout the match	11	1.00	4.00	2.18 (1.08)
Technical aspects of refereeing	11	1.00	4.00	2.00 (1.00)
Pre-match preparation	11	1.00	5.00	2.82 (1.17)
Refereeing philosophy	11	1.00	4.00	2.18 (0.98)
Self-confidence	11	1.00	4.00	1.91 (1.14)
Motivation	11	1.00	3.00	1.73 (0.79)
Pressure increase	11	1.00	3.00	1.55 (0.69)
Pressure decrease	11	1.00	5.00	2.45 (1.29)
Changes in own public perception	11	2.00	3.00	2.55 (0.52)
Changes in own Referee Union status	11	1.00	3.00	2.28 (0.79)

*VAR, video assistant referee.*

It was the same with the additional assistant referees (AAR)—when it started, everyone thought it’s a good thing that we have an extra pair of eyes. But then they began to acknowledge the problems (bad angle, fear of making decisions) and they started to drop this. Referees were afraid to be AARs. It’s the same process with the VAR.

The data presented in Figure 1 show that the referees perceived this change-event in the context of their careers with a moderate emotional profile. Specifically, they evaluated the significance of this change-event in their careers as moderate, its severity as neutral, and responded with moderate emotional and cognitive responses. Moreover, their perceived control over this new situation was also moderate. Expectedly, as the referees felt more control over the new situation, their cognitive reaction was less negative (i.e., less worried; *r* = −0.86, *p* < 0.01). Similarly, as past experience in coping with such change-events was higher, they also felt less worried (*r* = −0.75, *p* < 0.01). Finally, the higher was their age, the more positive was their emotional reaction (*r* = 0.60, *p* < 0.05).

### Demands and Barriers

Table 2 data revealed that the largest effects of the VAR system integration on refereeing were in pre-match preparation, players’ management, public perception, and DM—in this order. Interestingly, the referees felt that the pressure they experienced during the matches decreased rather than increased. Thus, it is evident that the integration of the VAR system did not create extreme professional demands for the Israeli referees. As one of them commented: “the change is mostly mental to cope with an error during the match. From a professional standpoint, the change is minimal and requires small and insignificant modifications.” Nevertheless, looking at the qualitative data, it seems that there were meaningful differences among the referees in the tone in which they related to this change process. While some referees perceived this change-event in a neutral manner, some perceived it favorably and others unfavorably. The following section, therefore, depicts the demands and barriers associated with this change-event, classified into off-field and on-field.

#### Off-Field Demands

The initial instruction of the VAR system (September 2018–March 2019) in terms of following the IFAB protocol was highly demanding in both time and energy. The referees were required to travel weekly to the instruction facility located in the center of Israel. They received some monetary reward for these educational sessions, yet not necessarily perceived as satisfactory for the time invested. They were informed, however, that the VAR integration would produce additional officiating options in the future, so the initial instruction investment was worthwhile.

During this period, which occurred in the active parts of the 2018–2019 season, the referees hardly received any regular refereeing instruction. This impacted their immediate performance, even before the actual application of the VAR system in live matches. From the project manager’s standpoint, the main challenge was to uncover the ideal balance between the necessary knowledge required for a successful training and keeping the tight schedule. It was clear that a 12-month period was much more realistic and suitable for such a complex training process than 6 months. To develop proficiency, the VARs are required to watch many match clips and acknowledge the correct level of intervention. The referees felt a strong sense of duty and responsibility to “deliver back the match” to the Referee Union. As one of them reflected: “there is zero room for errors in the VAR, while on-field errors get more tolerance.” Moreover, the VARs were somewhat “the protective shield,” making sure the matches came to a safe conclusion, without any club or public scrutiny. As one of them acknowledged:

The VAR is ungrateful, challenging. There is more pressure than on the field… I make decisions for someone else—it’s more complicated. There is a gap in the attitude (toward the match), and there’s a will not to screw the referee.

In addition, after several weeks in the 2019–2020 season, the referees were beginning to experience an officiating overload, as they were required to officiate as both head referees and as VARs in the same league round, in consecutive days. This created high mental demand; they lacked time to recover from one match and already were required to participate in another match.

Moreover, as the VAR is not only a professional refereeing system but also a social–ethical type of system, a need was raised to educate the “world of soccer” (i.e., the clubs, the teams, the media, the broadcasting staff, the commentators, and the fans and general public) about the new system; when and how it is being used. In this context, the Israel Referee Union was required to set the “correct” intervention level for the various types of match incidents in order to reach a degree of unity among all Premier League referees. The purpose was to create some balance between the existing UEFA intervention level and the one suitable for the domestic league. For example, in UEFA, the requirement is for minimum intervention (i.e., a very high intervention level), yet the Israeli clubs and media called for a low intervention level, as they wished to experience the system’s full influence. Thus, the Referee Union decided to publish after each league round the professional committee’s verdicts concerning the correct match decisions and the critical errors in which the VAR system was involved for better and worse. However, this did not reduce the pressure coming from the clubs and media when they perceived that an error was made. Alternatively, it stressed one of the main issues with the VAR system—that fairness is a relative term influenced by a sociocultural context. As one of the referees commented: “VAR is not an absolute justice machine, but a tool to prevent errors in critical decisions.”

#### On-Field Demands

Taking the VAR role, the referees were required to learn how to manually operate the video and audio systems, how to quickly and efficiently identify match incidents, when and how to interfere within the match, and how to effectively communicate with the on-field referees. They were also required to adapt themselves to sitting quietly in the VOR (i.e., a very small and somewhat claustrophobic location) for a long duration and manage their arousal and stress differently than they were used to (i.e., they could not just run to reduce their arousal levels like they typically do on the field). In this context, the referees identified professional demands such as being highly familiar with the VAR protocol, having patience and restraint, communicating correctly in the audio system, being knowledgeable of the Laws of the Game specific criteria, quick use of the video system, not getting disconnected from the match spirit, understanding the match, picking the correct camera angle, making correct decisions, and being a fast learner to close gaps as quickly as possible. It is important to note that when VAR intervenes, the DM is critical, which further increases pressure. However, mere accurate decisions might not be enough, as one of the referees explained:

In the van, you must have a macro vision—to be able to understand how this is being perceived by the public. Few low criteria are not sufficient; you must have lots of “meat” and a good reason for the decision to bring the story.

Another professional demand being a VAR was to be able to adjust to different referees in terms of level of intervention and communication style. Each referee expressed a different approach from the VAR, yet there were strict IFAB communication protocols. The VARs were not allowed to deliver certain information, which did not apply directly to their areas of responsibilities. Thus, serving as VARs, the referees were required to adjust between the demands of the specific head referee, the guidelines of the Referee Union professional committee, and their own refereeing philosophy to create the optimal level of intervention.

From the on-field referee standpoint, the referees must have learned the VAR protocol and accordingly adjust their refereeing. This meant modification of body language and new signing, as well as waiting after potential critical match incidents to hear from the VAR that the match could be continued. Also, they were required to manage a larger refereeing team, modifying their pre-match instructions to explain the level of intervention they were aspired to achieve. In addition, the referees were expected to adapt to having a “big brother” who watches their decisions and might intervene and correct them. They were required to adjust to a new situation in which every decision was not finite and could be corrected. Waiting for the VAR to check potential match incidents while being mobbed by the players was initially challenging, as the protocol was new for all stakeholders. They also must have adjusted to the OFR, which required them to go and check the match incident in the Referee Review Area (RRA) in a relatively short time period (i.e., up to 30 s is considered very good). In terms of DM quality, when the referee goes to the OFR, s/he shifts from an on-field DM to a video DM, which requires the referees to learn this new methodology. A further major demand was to first acknowledge an error in a critical decision and then to continue officiating the match with confidence and focus. Knowing that going to the OFR to correct a critical decision would result in a poor performance mark was also challenging.

#### Off-Field Barriers

There were also several *barriers* the referees experienced in their attempts to adapt to the VAR system. First, it is the relative lack of experience with the VAR system as well as the break formed in the implementation process (i.e., between the 2018–2019 playoffs and the beginning of the 2019–2020 season). Also, there were not enough VAR-related instruction clips, and the instructor lacked a definitive correct response for each VAR situation (i.e., unlike the typical refereeing instruction). This initially created frustration and reduced self-efficacy beliefs among the referees. As each country implemented the VAR system rather differently, it was difficult to learn much from the international colleagues’ experience.

The referees also mentioned the soccer world’s lack of understanding of the new system. In many cases, the referees performed according to the IFAB protocol, producing an accurate DM process (i.e., either to use the OFR or not, whether to modify their original decisions or not), yet still the soccer world perceived that the process to be incorrect or inaccurate. One of the referees commented on this issue:

There is much focus and screen time that goes to us now, and we must perform this in the most efficient way because the media and the whole soccer world would be much less tolerant toward us if we maintained an error even after we watched [on video].

In addition, the VAR learning process did not include a gradual sequence comprised of clear goals, levels of difficulty, rewards, and immediate feedback. This stemmed from the system being novel not only to the referees but also to the Israel Referee Union and to its professional committee. Thus, the referees were unsure, at the beginning, how the match marks of both the on-field and the VAR performances would be calculated and what would be the rewards and punishments for successful and poor performances. In fact, as the system was highly pressured by the Israeli soccer world, excessive attention was given to punishments and scrutiny, and the referees were hardly commended and rewarded for their efforts. This led to much frustration and, in some cases, to reduced motivation to officiate both on-field and as VARs.

Finally, there were also technical–technological issues in some of the stadiums and matches. Unlike the major matches, in the minor matches, there were only four cameras involved in broadcasting, thus certain important view angles were missing. In two stadiums, there were also issues with the system’s capacity to produce an accurate line grid of the field to enable valid video-based offside decisions. Also, in certain matches, the system could not be initiated on time, and the matches started without its use.

#### On-Field Barriers

From the on-field referee’s standpoint, several referees wished to officiate the matches without getting corrected by the VAR. Moreover, they did not accept the VARs’ corrections in certain cases and maintained their original decision. This had stemmed from the gaps in interpretation of key match incidents among the various referees. As one of them commented:

I feel that the VARs are not synchronized with my level of foul decisions on the field. I tell the VAR—“if it takes you a long time then it’s not clear.” If I see in the OFR what I saw on the field, then I would not change my decision.

The discrepancies between the on-field referees and the VARs had also stemmed from the fact that being called to the OFR and changing one’s decision resulted in a low match mark. Furthermore, once the referees were starting to contradict the VAR’s ruling, the Israeli media began to claim that the referees had interpersonal issues and “ego problems,” which affected their cooperation (Lipkin, 2020). As one of the referees commented: “every time that there is a wrong intervention of the VAR, it negatively affects the authority of the system.” In certain cases, there were repercussions on behalf of the Referee Union professional committee, with the referee getting suspended from officiating 1–3 matches. This had both financial and morale impact on the referees.

Certain referees also modified their performance to accommodate the new system in an undesired manner. For example, they issued cards and called penalties not according to their own perceptions but rather to how these events would be perceived by the VAR. Some referees even began taking less critical decisions on-field, knowing that the VAR would correct them if necessary. This was exemplified in the following quote:

VAR blurs the gaps between those who are good and those who are mediocre in refereeing. There are those who crash the matches and continue to officiate. You must encourage excellence and high skills. Now the situation is that referees run on the field just waiting for the VAR to make decisions for them.

In this context, certain referees had also begun stopping the match with every minor infringement to prevent “a problem” in the Attacking Possession Phase, in case of a potential VAR check. As one of them commented: “as a referee, you might change your officiating because of VAR—take decisions that prior to the VAR aera you wouldn’t take.”

In addition, there were significant gaps in interpretations between the referees and the professional committee. For example, the latter expected the referees to issue red cards in all “above ankle stamping” incidents, even if malice or excessive force was not evident (i.e., unlike what the official law criteria suggested). Certain referees, however, had not accepted these instructions, and when called to the OFR did not modify their original decisions. These actions, in turn, resulted in the referee being suspended. These incidents further intensified the lack of clarity as well as undermined the referees’ belief in the new system: “in the OFR, I go into a dilemma—to stay with the red card that might not be an error; to change or not? The conflict is loss vs. profit.”

There were also errors of the VARs. These stemmed from ineffective communication with the on-field referee, low skills with the video system, as well as performance anxiety. As one of the referees commented: “the errors stem from the VARs’ desire to please the on-field referee, to not just call for the OFR for a long-time… and there is a lack of skill.” In this context, the professional committee suspended VARs who made critical errors. This frustrated some of the referees, as reflected in the following quote:

A freaky method in which they publicly suspend referees during the system’s implementation process. This promotes “tricks” and unprofessional mode of actions on behalf of the referees… they had to decide ahead of time what would be the suspension method in cases of VAR errors.

### Decisions and Coping

When the referees were firstly informed of the upcoming integration of the VAR, they were already aware of its effects in other countries. Thus, as part of their initial response to the new situation, nine of the referees decided to consult with others, mainly with colleagues, a refereeing mentor, the professional committee and the Referee Union, and a sport psychologist. Two of the referees decided to self-cope as part of their initial strategy. One of them explained that “initially, I tried to figure out what this means, evaluate the new situation, and check how much the world of refereeing is about to change.” For another referee, “it was a challenge to be successful with the VAR system.” Furthermore, nine of the referees made a decision to change (i.e., apply all necessary adjustments in response to the new situation). For example, one of them commented: “I contemplated and realized the changes and then “hugged” [the new system] as the right thing to do with many advantages that can assist me.” Two of the referees decided to consult with others as their main coping strategy. This also reflected in their comments: “stick to the guidance of the professional committee; tips and recommendations of the sport psychologist.”

Referring to Figure 1, it seems the referees maintained high motivation for change. Still, their effectiveness and satisfaction of coping were only moderate. They also felt that their experience had moderately facilitated their current coping efforts. There was a positive association between the helpfulness of experience in similar situations and the current coping effectiveness, *r* = 0.70, *p* < 0.05.

The referees reported various types of coping strategies, mostly adaptive: accepting the VAR system as a fact, taking advantage of the its benefits, consulting with professional resources, reevaluation of the refereeing role—“we are here to serve the teams and justice must be seen,” mental preparation for matches, adapting oneself to the professional guidelines, becoming highly knowledgeable of the VAR protocols, watching many VAR clips, self-analysis of VAR performance and communication, and live performances.

### Support Resources

Data from Figure 1 suggest that the referees experienced high availability of professional support resources (e.g., sport psychologist, professional mentor, and refereeing coach). They also rated the helpfulness of the sport psychologist and professional mentor as high. Nevertheless, they rated their degree of cooperation with the professional committee as moderate. On one hand, the professional committee was responsible for providing the referees with the technical guidelines related to the VAR application. On the other hand, it monitored the referees’ performances and sanctioned them when errors were made. This had created a degree of mistrust between the referees and the professional committee. The referees’ cooperation with the professional committee correlated positively with the effectiveness of their coping (*r* = 0.62, *p* < 0.05). This indicates the importance of a positive cooperation in such a change-event, which involves new instructions and technical adaptation.

### Change Process Outcomes

**FIGURE 2:**
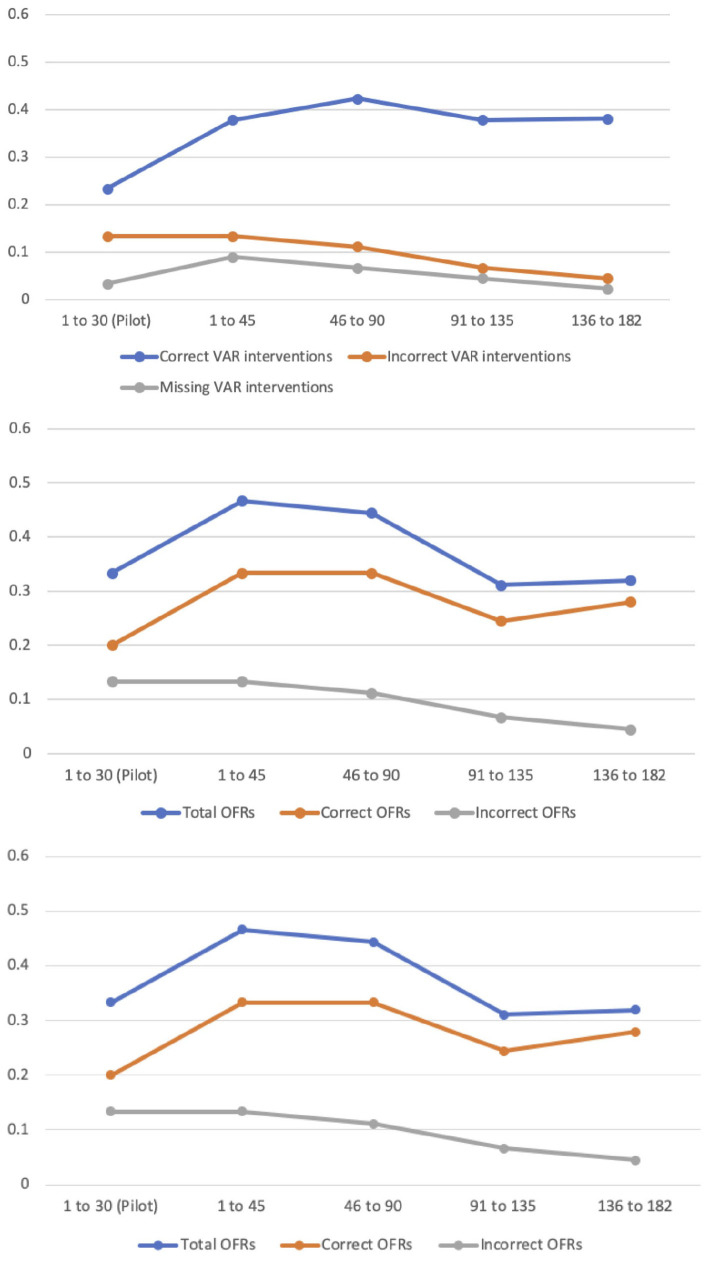
Per match frequency count of Overall Critical Errors and Rectified On-Field Critical Errors **(upper panel)**; Correct Video Assistant Referee (VAR) Interventions, Incorrect VAR Interventions, and Missing VAR Interventions **(middle panel)**; and Total On-Field Reviews (OFRs), Correct OFRs, and Incorrect OFRs **(lower panel)**.

Frequency counts (per match) were computed for all variables of interest describing the referees’ performance with the VAR system (Figure 2). The frequency of *Overall Critical Errors* was higher for Matches 1–45 compared to the pilot phase of the program (Matches 1–30); χ^2^(3) = 20.35, *p* < 0.01. The number of *Correct VAR Interventions* was also higher for Matches 1–45 compared to the pilot phase of the program (Matches 1–30); χ^2^(3) = 18.92, *p* < 0.01. Hence, the increase in *Overall Critical Errors* for Matches 1–45 was positively balanced by an increase in the number of *Correct VAR Interventions* for these same matches. No other statistical effects were observed, but the overall trend for all variables was positive over time (Figure 2). Specifically, Incorrect VAR Interventions, Missing VAR Interventions, and Incorrect OFRs showed the lowest values at the last phase of the program (Matches 136–182). *Total OFRs* and *Correct OFRs* increased after the pilot phase of the program and showed a mirrored fluctuating pattern afterward (Figure 2). Therefore, as the referees became more proficient with the VAR system, there was some improvement in performance. Also, the average match time suspension due to VAR interventions decreased as the season progressed, further indicating the referees’ technical and DM proficiency. Nevertheless, these were small improvements not always recognized by the soccer world and the referees themselves.

On average, the referees perceived the outcome of this change-event as relatively neutral in the context of their careers (Figure 1). Yet, looking at the variance, it is evident that three referees perceived it positively, four perceived it neutrally, and four negatively. The referees were also moderately satisfied with their match performances both on-field and as VARs. Moreover, a positive correlation between the referees’ satisfaction from their on-field performances and the perceived outcome of this change-event, *r* = 0.67, *p* < 0.05, emerged. This might indicate that the referees were more concerned about the effects of the VAR system on their on-field performances. Finally, their motivation for refereeing was high and remained so even after the VAR implementation (Figure 1).

## Discussion

With the growing recognition of soccer referees as performers on their own merit (Aragão e Pina et al., 2018), research attention was given to various aspects of their roles, including career development (Samuel et al., 2017). The introduction and implementation process of the VAR system within the Israeli Premier League reflected a technical–technological change-event (i.e., a quasi-normative transition) in the careers of the Israeli elite referees. This change-event introduced potential implications for training, performance, public status, and professional advancement (Armenteros and Webb, 2020; de la Vega and Fuentes, 2020).

Based on Kolbinger and Lames (2017) taxonomy, there are technologies that support the DM process of referees. Technologies are used to replace referees for a specific decision, and technological aids help the referee to enforce rules (e.g., the vanishing spray in soccer). The VAR system is supposedly related to the first classification, as it was designed to improve referees’ DM by offering additional view angles as well as video replays. However, it might also set conditions for replacing the referee in certain basic decisions, such as offsides and goals. Furthermore, knowing that they are being watched and supervised by the VAR, the players are committing less fouls (Lago-Peñas et al., 2019), supporting the third classification as well.

The official data concerning the implementation of the VAR system in Israel indicated that, in 212 matches, there were 89 critical errors in total. This rate was higher than that in previous years, which could be attributed to the introduction of the VAR and the detection of more key match incidents, as well as to lower on-field refereeing quality due to the adaptation process. The initial use of the system was far from perfect, with error rates being higher than expected. In the Italian implementation phase, for comparison, the reported level of error was 1%, with 1,708 decisions being reviewed over 210 matches, which led to 60 decisions being corrected, 11 of those being wrong, including seven that influenced the outcome of the game (Simón, 2020).

The Israeli Premier League referees perceived the VAR implementation as a career change-event characterized by a moderate emotional profile. This change-event had mainly influenced the technical–technological aspect of their performance, with moderate effects on pre-match preparation, players’ management and communication, public perception, and DM. These areas of refereeing might be associated with the referees’ authority and credibility. Previous research suggested a potential threat of technological officiating aids to referees’ authority (Kolbinger and Lames, 2017). Nevertheless, the effects on career development were minor. Moreover, research on referees’ careers indicated that change-events related to career development were perceived as highly significant, whereas performance-related issues were perceived moderately (Samuel et al., 2017; Samuel, 2019). Also, a recent study on the regulations and refereeing modifications in competitive judo showed that the judokas and coaches perceived them with a moderate emotional profile (Samuel et al., 2020a). Thus, the findings of the present study are in line with research indicating that technical–technological modifications are being perceived with a moderate emotional profile, with modest effects on referees’ performance as well as authority. These findings, therefore, provided support for the application of the SCSPP (Samuel and Tenenbaum, 2011a) in the context of referees’ careers.

In addition, the emotional and cognitive perceptions of the referees were associated with their perceived control, their experience with similar situations, as well as their age. It seems that as the referees were older and more experienced, they perceived this change-event more favorably. In a sample of Swedish soccer referees, Folkesson et al. (2002) found that younger referees were shown to be the most prone to threat and aggression. It is possible that the younger Israeli referees in the present study were also more apprehensive of their ability to effectively manage players and make accurate decisions upon VAR implementation.

The findings indicated that most off-field demands were related to the instruction and operation of the VAR system. This change-event required the referees to quickly and efficiently integrate a new method of refereeing, accepting its importance for both the soccer world and the Referee Union. Fernández Ruiz et al. (2020) advised that as part of developing the VAR education curriculum, “learning activities for referees can be distributed over time, and with the aid of learning management systems and other applications, instructors can control this distribution, programming specific dates and completion times for the activities” (p. 337). However, in the Israeli case, the 12 months’ IFAB protocol was shortened by half, presenting high pressure and demands. The referees were, therefore, under considerable workload while learning the new system and then attempting to adjust to it. They were asked to significantly increase the amount of active officiating in each league round, which further led to overload. This, in turn, resulted in lower refereeing quality and criticism from the Referee Union professional committee. These findings echo Baldwin’s (2014) study of Australian rugby league referees, who reported much stress related to the education of the video replay technology.

Another important demand was to educate the soccer world about the new system. Although there was a standard IFAB protocol, each country implemented the VAR system somewhat differently (Armenteros and Webb, 2020). At first, the Israeli soccer world expected the VAR system to intervene in all decisions, which was not in line with the IFAB protocol. This created pressure on the referees and the professional committee. Thus, the referees and the VAR system were under much scrutiny in terms of what Kolbinger and Lames (2017) identified as *standard of review*—the influence of the initial call of the referee on the review process and its outcome.

In terms of the on-field demands, the referees were required to quickly learn a new method of officiating as VARs, which is significantly different from their typical on-field refereeing task. Adapting to this new system required forming a unique man–machine interface as well as developing specified DM skills. Fernández Ruiz et al. (2020) suggested that referees must apply 21st century skills (e.g., technical, information management, and critical thinking) while performing the VAR task. In this context, it is unclear what was the referees’ degree of *digital competence and efficacy* (Janssen et al., 2013) when faced with the technological demands. Future research must, therefore, focus on analyzing the most effective DM process for the on-field and VAR refereeing task, considering the quick transformations between sequential DM and video-based DM. A critical aspect of these transformations is how to include the contextual match aspects to achieve not only correctness but also fairness (Samuel et al., 2020b).

Moreover, the referees were expected to accept that a “big brother” was watching their decisions, not necessarily intending to overrule them, but rather to support and assist them. This required a major shift in the referees’ mindset. Samuel et al. (2020b) proposed that, in the final phase of the DM sequence, referees may decide to keep or change their decision. As previous research indicated, referees are individuals with high trait and state self-control (Samuel et al., 2018). Thus, asking them to incorporate the VAR into their on-field refereeing meant trusting the VARs’ professional quality and personal integrity. This required “a leap of faith,” that at least at the beginning of this process was highly demanding for most of them. Research findings indicated that *making a controversial call* and *making a wrong call* are considered high stressors for soccer referees (Voight, 2009), potentially leading to increased anxiety (Yun and Jeon, 2016). As the elite refereeing environment is highly competitive (Samuel et al., 2017), the referees were apprehensive about getting corrected by the VARs. Still, they reported that their pressure decreased as a result of the VAR integration, which might indicate that they preferred to end a match with a rectified critical error than letting such an error remain.

The barriers involved in this change-event corresponded to the on- and off-field demands. The off-field barriers included the educational process, gaps in interpretations with the world of soccer, lack of clear goals and rewards system, and technical–technological setbacks. All these barriers were previously identified in other VAR implantations (e.g., Baldwin, 2014; Fernández Ruiz et al., 2020; Kolbinger, 2020; Simón, 2020). For example, in the German Bundesliga VAR implementation, the head of the elite referees committee, Lutz Michael Fröhlich, concluded after the first five match days that “a lot of players and team officials still struggle to differentiate between apparently similar situations, which are in fact different and therefore must be treated differently by the VAR protocols” (Kolbinger, 2020, p. 233). Thus, discrepancies in interpretations concerning the application of the VAR were evident in other leading countries, not only in Israel.

The on-field barriers included gaps between the referees and the VARs, modifications of the referees’ typical officiating style, gaps between the referees and the professional committee, and errors of the VARs. Some of these barriers were also previously identified (Baldwin, 2014; Armenteros and Webb, 2020; Bacigalupe, 2020). For example, the German elite referee committee decided that “for difficult calls, which can’t be rated indisputably as ‘obvious mistakes,’ but the video assistant doubts the initial decision by a high degree, he shall immediately contact the referee to initiate an on-field review” (Kolbinger, 2020, p. 233). This quote suggests some gaps in interpretations between the German referees and the professional committee. Furthermore, Bacigalupe (2020) proposed that the VAR is under pressure “as they are the watchdog—and therefore ultimately responsible—who is there to make sure nothing that happens on the pitch goes unseen” (p. 201). Unlike the on-field referee who is given some latitude when erring, there is no tolerance for VAR errors. A challenge is not to fall into a trap of justifying the on-field referee’s decisions.

There were also unique barriers for the Israeli referees. For example, the referees’ lack of adherence to the VARs’ calls, perhaps due to match mark interests or to ego-protecting interests, was not discussed in the literature so far. These motives are typical for the highly competitive Israeli elite referees (Samuel et al., 2017). In this context, the system of rewards for VAR officiating was unclear, with high focus on sanctions and scrutiny and too little praise. For almost all referees, this led to a negative motivational process and a heightened fear of failure (Samuel, 2019).

Also, the modification of officiating style has not been recognized by researchers yet. The Israeli referees modified their regular style both on-field and as VARs to decrease potential errors and increase their level of accountability. Once more, these effects are related to the unique Israeli context where the referees did not wish to be criticized by the professional committee as well as to sustain lower match marks.

Even though this change-event was not perceived as highly demanding, it required coping efforts. Most referees made active decisions as part of their coping, initially to consult with professional resources and subsequently to apply all necessary changes to effectively cope. These decisions manifested in application of adaptive coping strategies. These findings are in line with the literature on career change-events in referees (e.g., Samuel et al., 2017; Samuel, 2019). Thus, it seems that the referees assumed an active approach in their attempts to adapt to the VAR system. In addition, while the referees felt they have had high availability of professional support, their cooperation with the professional committee was lacking, negatively impacting the effectiveness of their coping. The professional committee had a dual role in the VAR implementation process, which included (a) establishing contextualized implementation standards for the Israeli league (i.e., an educational role) and (b) regulating misapplications of the system (i.e., a punitive role). These findings, therefore, emphasize the role of the domestic professional committees in providing the right support for referees who undergo a technical change. This support should be manifested in setting clear goals for the change process, being tolerant toward referees’ errors and confusions and protecting the referees from scrutiny of the media and other stakeholders.

In terms of the change process outcomes, the Israeli referees showed small, yet important, improvements in their performance. However, they did not receive credit for them. On the contrary, in many cases, they experienced scrutiny from the clubs, the media, and the Referee Union’s professional committee. Furthermore, the referees varied in their perception of this change process outcome, with three perceiving it positively, four neutrally, and four negatively. These findings further demonstrate the dynamic and probabilistic nature of the change process (Stambulova and Samuel, 2020). An important factor was their degree of satisfaction from their on-field performance since the VAR was integrated. This finding is on a par with Samuel et al.’s (2020a) study on the regulations and refereeing modifications in competitive judo; the judokas’ perceptions of the outcome of that change-event were associated with their perceptions of professional achievements since these modifications took place. These findings are important, as they explain to the referee unions that, in the end, the VAR is here to serve the on-field refereeing, not the other way around. Referees are motivated to officiate the matches on the field, applying their long-learned skills and experience. Finally, the referees maintained high motivation following the VAR integration into the domain of refereeing.

To conclude, much of the above findings are in line with the emerging literature on technological officiating aids (Kolbinger and Lames, 2017; Samuel et al., 2020a), including VAR (Armenteros et al., 2020), as well as with the literature on referees’ change-events (Samuel et al., 2017; Samuel, 2019). Generally, these findings emphasize that implementing the VAR presents a moderate change-event in soccer referees’ careers that require a degree of adaptation in terms of active DM, coping, and support.

### Study Limitations

We must consider limitations to the current study. First, while the sample was highly representative of the entire Israeli Premier League squad, not all referees participated in the study. Those who responded received psychological support, and it is possible that this had influenced their change process. Thus, sampling referees who do not receive psychological support throughout the VAR implementation is imperative. Also, the study is highly contextualized within the Israeli soccer culture. While contextualizing career-related studies is called for (Stambulova et al., 2020), it might also influence the potential for generalizing the findings to other populations. Moreover, all participants were males, and gender effects were not examined. Thus, examining how VAR is perceived and implemented in other countries and refereeing populations is of interest, while adopting an empathetic stance and realizing the particularities of each country. Also, there might be other potential factors not examined in this case study that might influence referees’ perceptions of change processes, such as referees’ salaries and monetary and professional rewards (Fernández Ruiz et al., 2020), as well as digital competence (Janssen et al., 2013). Finally, we did not collect quantitative data in several time points, as conducted in previous change-event studies (e.g., Samuel et al., 2015). It is possible that collecting data after a longer time period following the VAR integration would allow a different perspective on its effects on referees’ careers. Future studies that accompany referees’ change process are advised to apply a full longitudinal design. Also, as there were several CEI subscales with low reliability indices, related correlational data must be cautiously viewed.

### Practical Recommendations

Referee unions should provide referees with the adequate support to be able to respond to this change favorably. First, to acknowledge the importance of the VAR and its correct role—not to replace on-field officiating, but to assist it. Second, to recognize the key refereeing areas, which such a change might impact. In the present case, the largest effects of the VAR system integration were in pre-match preparation and players’ management. These are two areas, as well as DM, in which referees can progress, given the appropriate training support (e.g., Cunningham et al., 2014; Samuel, 2015). Still, referees in other countries might experience modifications in additional areas. It is advised to construct a gradual educational process, not as intense as in the Israeli case. This could be optimized by setting a clear system of goals, providing ample feedback, establishing rewards and sanctions, and considering the importance of praise over scrutiny. In this context, we recommend referee unions to allow referees a continued training process following the IFAB certification. This would entail the development of novel training modalities, in addition to existing VAR simulators, so referees can continue independent practice. New online options, such as Zoom-based training (see Samuel et al., 2020c), can be exploited for this purpose. Analyzing the refereeing team communication in critical match incidents is of high importance. Relying on a clear statistical dataset to assess individual and collective progress of VAR-related skills is also important. Psychological support must focus on accepting the change and recognizing its impact both on- and off-field. Then, making a conscious decision to change and applying it in the ordinary refereeing routine. Finally, as VAR is not only a professional system but also a social–ethical one, much consideration should be given to education of the soccer world concerning its application. The clubs, fans, and media must be more knowledgeable of how and when the system is used to produce more correct, just, and fair decisions.

## Data Availability Statement

The datasets for this article are not publicly available due to the high profile of participants and confidentially of data. Requests to access the datasets should be directed to RS, roydsamuel@gmail.com.

## Ethics Statement

Ethical review and approval was not required for the study on human participants in accordance with the local legislation and institutional requirements. The patients/participants provided their written informed consent to participate in this study.

## Author Contributions

RS wrote the literature review. RS and EF wrote the methods and findings. RS, YG, EF, and GT wrote the conclusion and discussion. All authors contributed to the article and approved the submitted version.

## Conflict of Interest

The authors declare that the research was conducted in the absence of any commercial or financial relationships that could be construed as a potential conflict of interest.
